# Cotton *STARD* Gene Family: Characterization, Evolution, and Expression Profiles During Salt Stress

**DOI:** 10.3390/genes16070813

**Published:** 2025-07-11

**Authors:** Ruifeng Cui, Jiuguang Sun, Shuyan Li, Yupeng Cui, Cun Rui, Minshan Sun, Wuwei Ye

**Affiliations:** 1 College of Biology and Food Engineering, Anyang Institute of Technology, Anyang 455000, China; xiaocui0126@126.com (R.C.); sunjiuguang@126.com (J.S.); lishuyan6688@163.com (S.L.); yupeng_cui851026@163.com (Y.C.); rc1728891526@126.com (C.R.); 2 College of Plant Protection, Henan Agricultural University, Zhengzhou 450002, China; 3 Anyang Institute of Technology/Research Base, State Key Laboratory of Cotton Bio-Breeding and Integrated Utilization, Institute of Cotton Research of Chinese Academy of Agricultural Sciences/National Center of Technology Innovation for Comprehensive Utilization of Saline-Alkali Land, Anyang 455000, China

**Keywords:** cotton, *STARD*, gene family, salt stress, gene expression

## Abstract

**Background**: Cotton, a key global economic crop, suffers yield and quality losses due to salt stress. This study aims to analyze the cotton *STARD* gene family and its role in salt stress responses. **Methods**: We conducted a genome-wide analysis of the *STARD* gene family in four cotton species, using phylogenetic trees, chromosomal mapping, and collinearity analyses to explore their evolutionary relationships and expansion mechanisms. We also examined gene structures, conserved motifs, and promoter *cis*-elements. **Results**: *STARD* genes are evenly distributed across the four cotton species. Segmental duplication was found to be the main driver of gene expansion, with most pairs undergoing purifying selection. Distinct structural features and potential roles in plant growth and stress responses were identified. Notably, 11 *GhSTARD* genes showed significant expression changes under salt stress, especially *GhSTARD45* in root tissues. **Conclusions**: This study provides new insights into the function and salt stress response mechanisms of the cotton *STARD* gene family, suggesting *GhSTARD45* plays a key role in root-mediated salt tolerance and highlighting the potential of *STARD* genes in enhancing cotton’s salt tolerance.

## 1. Introduction

The STARD protein family members contain a conserved sequence of approximately 210 amino acids, with their core structure being the *α*/*β* helix-grip fold [1,2]. Initially discovered in mammals, their hydrophobic cavity can bind lipid ligands and small molecular modules (such as cholesterol, phospholipids, etc.) and mediate lipid transport and signal regulation through non-vesicular pathways [3,4]. This family can be classified into five categories based on domain combinations: HD-START, HD-START-MEKHLA, PH-START-DUF1336, START-DUF1336, and independent START domains [5]. Notably, transcription factors of the HD-Zip gene family (such as HD-Zip III and IV subgroups) are also categorized as STARD members. The HD-Zip III subgroup includes START, HD, and MEKHLA domains, while the HD-Zip IV subgroup contains only START and HD domains [6,7,8].

In plants, STARD family members are extensively involved in growth, development, and stress responses: the *Arabidopsis* HD-Zip IV subgroup member GL2 regulates trichome development through the HD-START domain, while HD-Zip III subgroup members ATHB-8 and REV participate in vascular bundle differentiation and leaf polarity establishment, respectively [7,8,9,10]. Furthermore, the heterologous expression of *Arabidopsis HDG11* (HD-Zip IV) in rice significantly enhances drought tolerance and yield. *Arabidopsis AtAPO1* and *EDR2* regulate cytoskeletal organization and defense responses via PH-START domains, and *AtAPOSTART1* affects seed germination [11,12]. In the plant pathogenic oomycete *Phytophthora capsici*, the ORP1 protein relies on PH and START domains for subcellular localization, and its deletion leads to pathogen death, suggesting these domains as potential fungicide targets [13,14]. The function of the DUF1336 domain at the carboxy terminus of PH-START proteins remains unclear, but in Phytophthora sojae, it is associated with pathogenicity and may participate in host interaction or lipid metabolism regulation [15].

Recent studies have revealed the dual potential of STARD proteins in plant stress resistance and pathogen control: maize *ZmSTARD1* enhances drought tolerance by binding abscisic acid (ABA), while wheat *TaSTARD7* maintains cell membrane stability under salt stress by regulating lipid metabolism [16]. The ORP1 protein of Phytophthora infestans hijacks host lipid signaling through the START domain, and small molecule inhibitors targeting this domain can block its infection process [17]. Similarly, the PH-START protein deletion mutant of *Pseudoperonospora cubensis* loses pathogenicity, providing a molecular target for novel fungicide design [17,18]. In grapes, researchers observed that the expression of STARD genes was induced by salt stress and MeJA treatment. Specifically, *VvSTARD5* overexpression resulted in reduced levels of malondialdehyde (MDA) and other stress markers following 24 h of NaCl treatment. Findings from these studies suggest that STARD proteins confer enhanced tolerance under plant stress conditions and may play a critical role in plant defense against abiotic stresses [19].

Cotton provides commercially valuable fibers essential for the textile, paper, and pharmaceutical industries [20,21]. Salt stress severely impacts cotton by reducing fiber yield and quality, posing a significant economic threat [22,23,24]. This study systematically characterized the STARD gene family across four cotton cultivars using the latest genome assembly, identifying 46–93 STARD genes per variety. Bioinformatic analyses revealed gene structures, subcellular localization predictions, and chromosomal distributions. Phylogenetic and motif analyses, combined with comparative genomics, classified these genes and identified conserved motifs. Evolutionary relationships were explored through collinearity and selective pressure analyses, while promoter analyses have predicted potential transcription factor interactions. Transcriptomic data mining identified salt-responsive *GhSTARD* candidates, validated by qRT-PCR showing tissue-specific expression of *GhSTARD45*. Protein–protein interaction networks further predicted functional associations. This work provides a foundation for cotton STARD research and genetic resources for improving salt tolerance.

## 2. Materials and Methods

### 2.1. Identification of STARD Genes in Cotton

Genome assemblies for *Gossypium arboreum* (CRI v1.0), *Gossypium raimondii* (JGI v2.0), *Gossypium hirsutum* (HAU v1.1), and *Gossypium barbadense* (ZJU v1.1) were obtained. The *Arabidopsis thaliana*, *Theobroma cacao*, *Vitis vinifera* and *Oryza sativa* genome was obtained from Phytozome (https://phytozome.jgi.doe.gov/pz/portal.html, accessed on 10 March 2025). HMM profiles for (PF01852) START domains were downloaded from Pfam (https://pfam.xfam.org/accessed on 10 March 2025). HMMER v3.0 software (http://www.hmmer.org/) was employed with an e-value threshold of 1 × 10^−5^ to identify candidate STARD proteins containing these domains. Putative STARDs were validated via Pfam motif scanning for further analysis. Transmembrane domains were predicted using TMHMM Server v2.0 (http://www.cbs.dtu.dk/services/TMHMM/, accessed on 10 March 2025 ). Biochemical parameters (pIs, MWs, GRAVY scores, charges) and exon–intron structures of G.hirsutum STARDs were extracted from CottonFGD. The subcellular localization of GhSTARD proteins was predicted using WOLF-PSORT (https://wolfpsort.hgc.jp/) [25].

### 2.2. Sequence Alignment and Phylogenetic Analysis

Full-length amino acid sequences of STARDs from *G. hirsutum*, *G. arboreum*, *G. raimondii*, *G. barbadense*, and other plants were aligned with ClustalW (https://www.genome.jp/tools-bin/clustalw, accessed on 10 March 2025) using default parameters. Maximum likelihood (ML) phylogenetic trees were constructed in MEGA X (Version 10.1.8, accessed on 10 March 2025) with 1000 bootstrap replicates under the Poisson model and visualized using EvolView (https://www.evolgenius.info/evolview, accessed on 12 March 2025) [26,27].

### 2.3. Chromosomal Locations of STARDs from Four Types of Cotton

Genomic, CDS, and GFF data for all four cotton species were obtained from CottonFGD. Physical chromosome positions of STARDs were mapped using TBtools (V1.098) [28].

### 2.4. Gene Duplication and Synteny Analysis in Different Cotton Types

Synteny blocks among *G. hirsutum*, *G. arboreum*, *G. raimondii*, and *G. barbadense* were identified using MCScanX [29] and visualized with Circos (http://circos.ca/, accessed on 10 July 2021) [26].

### 2.5. Calculation of Selection Pressure

Homologous gene pairs across the four cotton species were defined using MCScanX [29]. Selection pressure was assessed by calculating Ka/Ks ratios with KaKs_Calculator 3.0 [30].

### 2.6. Analysis of the Conserved Protein Motifs and Gene Structure

Gene structures (exons/introns) of *GhSTARDs* were visualized using GSDS (http://gsds.gao-lab.org/). Conserved protein motifs were identified via MEME with the following parameters: motif width 6–200 residues, maximum motifs = 20, and distribution = 0/1. The results were integrated with phylogenetic trees and G.hirsutum GFF3 data using TBtools [28].

### 2.7. Analysis of STARDs Promoter Regions

Promoter sequences (2000 bp upstream of TSS) of *GhSTARD*s were extracted from CottonFGD. *Cis*-regulatory elements were predicted using HOMER (http://homer.ucsd.edu/homer/) [31].

### 2.8. Gene Expression Pattern Analysis

Expression data were normalized using FPKM. *GhSTARD*s with FPKM > 1 or log_2_ (1 + FPKM) > 1 in the ≥1 stage were considered expressed. Tissue specificity was analyzed using a Python (Version 3.11.3, accessed on 10 March 2025) script. Heatmaps were generated using pheatmap.

### 2.9. RNA Extraction and RT qPCR Analysis

Total RNA was extracted with a Solarbio Kit (R1200) and reverse transcribed using PrimeScript™ II (TAKARA). RT-qPCR was performed with Vazyme Taq Pro Universal SYBR qPCR Master Mix (Q712-02) on a CFX96 system [32,33]. Data were analyzed via the 2^−ΔΔCT^ method using GhHis3 as the reference. Primers were designed with Primer3 and synthesized by Sangon Biotech [34]. Two cotyledon with one true leaf stage seedlings of upland cotton (*G. hirsutum*) varieties were subjected to the following treatment. The NaCl solution used had a concentration of 200 mmol/L. The seedlings were initially cultivated in a growth chamber using a 1:1 mixture of nutrient soil and vermiculite as the growing medium. The growth conditions in the chamber were set at 28 °C during the day and 25 °C at night with a 12 h photoperiod. For the treatment, the seedlings were gently removed from the growing medium by soaking them in water, and their roots were thoroughly rinsed to clean off any remaining soil. Excess moisture was then blotted away with absorbent paper. The cleaned seedlings were subsequently placed in conical flasks, with the liquid level in the flasks set to rise above the roots of the seedlings.

### 2.10. Protein–Protein Interaction (PPI) Analysis

To decipher the functional interactions among candidate genes, protein–protein interaction (PPI) networks were constructed using the STRING database (v12.0) (https://cn.string-db.org/). Relevant gene-encoded proteins were submitted to STRING’s “multiple proteins” mode, employing default parameters.

## 3. Results

### 3.1. Identification of the STARD Proteins in Cotton

In this study, we identified 93, 93, 48, and 46 full-length *STARD* family genes from *G. hirsutum* (allotetraploid cotton), *G. barbadense* (allotetraploid cotton), *G. arboreum* (diploid cotton), and *G. raimondii* (diploid cotton), respectively. A structural analysis confirmed that the STAR-AP subfamily proteins contain a conserved STARD-trans domain, while the STARC subfamily sequences feature one or more STAR_permease or STAR_permease_2 motifs. Notably, the CAT subgroup within the STARC subfamily uniquely possesses a STAR_permease_C signature domain [35]. The identified STARD members across the four cotton species were systematically named based on their chromosomal positions. For cross-species comparability, *GhSTARD* genes were additionally annotated according to their phylogenetic relationships with *A. thaliana*, *T. cacao*, *V. vinifera*, and *O. sativa* homologs. Prefixes *Gh*, *Gb*, *Ga*, *Gr*, *At*, *Os*, *Tc*, and *Vv* denote genes from *G. hirsutum*, *G. barbadense*, *G. arboreum*, *G. raimondii*, *A. thaliana*, *O. sativa*, *T. cacao*, and *V. vinifera*, respectively, with detailed annotations provided in Table S1.

The comprehensive characterization of *G. hirsutum* STARD members included genomic architecture metrics (genomic length, CDS length, and chromosomal localization), protein properties (amino acid length, isoelectric point, and molecular weight), and structural features (transmembrane domains). As shown in Table S1, the GhSTARD proteins exhibited substantial size variation, ranging from 114 residues (*GhSTARD5* on Ghir_A02) to 980 residues (*GhSTARD37* on Ghir_A10). The calculated isoelectric points spanned from 5.33 (*GhSTARD30*) to 9.68 (*GhSTARD4*), while the molecular weight was between 12.2 (*GhSTARD5*) and 93.78 (*GhSTARD37*). Subcellular localization predictions unanimously indicated that all GhSTARD proteins are distributed in several locations, including the chloroplast, cytoplasm, extracellular space, nucleus, vacuole, and plasma membrane-related or cytoplasmic fluid (Table S1).

### 3.2. Phylogenetic Analysis of STARD Gene Family in Cotton

**Figure 1:**
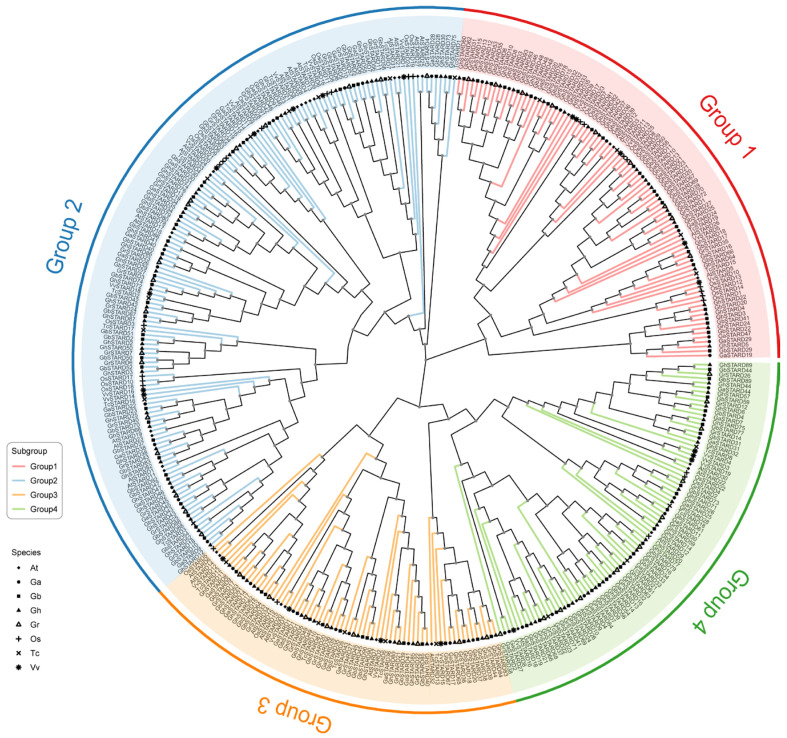
A phylogenetic tree depicting the evolutionary relationships among STARD proteins from cotton species (*Gossypium hirsutum*, *Gossypium barbadense*, *Gossypium arboreum*, and *Gossypium raimondii*) and STARD genes from *Arabidopsis thaliana*, *Oryza sativa*, *Vitis vinifera*, and *Theobroma cacao* was constructed. The tree shows STARD proteins grouped into four major clades (Groups I–IV), each represented by a distinct color. Different species are denoted by specific symbols: solid triangles (*Gossypium hirsutum*—*Gh*), hollow triangles (*Gossypium raimondii*—*Gr*), solid circles (*Arabidopsis thaliana*—*At*), hollow circles (*Oryza sativa*—*Os*), solid squares (*Vitis vinifera*—*Vv*), and hollow squares (*Theobroma cacao*—*Tc*).

To investigate the evolutionary relationships of STARD genes in cotton, we performed a multiple sequence alignment of all STARD protein sequences from *Arabidopsis thaliana*, *Oryza sativa*, *Theobroma cacao*, *Vitis vinifera*, and cotton using the maximum likelihood (ML) method in MEGA X software. A rooted phylogenetic tree was subsequently constructed. Based on existing research on the STARD family in other organisms, STARD members were classified into four major subfamilies. Group 1 comprises 89 genes, Group 2 contains the highest number of genes (147), Group 3 has the fewest (68), and Group 4 includes 80 genes. Within these four groups, the distribution of STARD genes across the four cotton species *Ga*, *Gr*, *Gb*, and *Gh* is nearly uniform. In Group 1, which has the fewest genes, there are 9, 9, 16, and 16 genes from *Ga*, *Gr*, *Gb*, and *Gh*, respectively, whereas in Group 4, which has the largest number, there are 14, 17, 34, and 34 genes from these species (Figure 1). Furthermore, we calculated the correlation of *STARD* gene numbers across species within each subgroup. The average correlation across different groups was found to be 0.95, indicating a relatively stable evolution of *STARD* genes in each species.

### 3.3. Chromosomal Localization of Cotton STARD Genes

**Figure 2:**
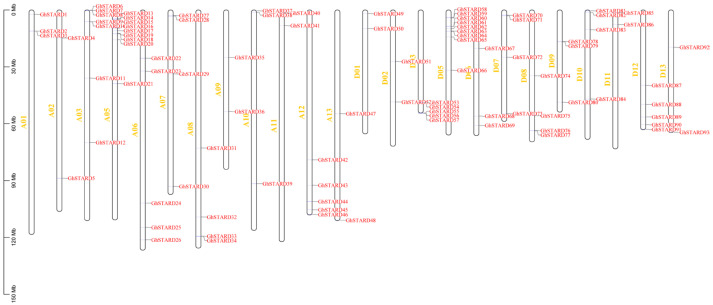
Distribution of *STARD* genes in *Gossypium hirsutum*. The y-axis indicates chromosome length, with chromosome numbering labeled in yellow font. Red markers denote the identified locations of *STARD* genes in *G. hirsutum*.

To elucidate the genomic organization patterns of STARD family genes in cotton, we conducted comprehensive chromosomal mapping of 280 STARD members across two allotetraploids (*G. hirsutum* and *G. barbadense*) and two diploids (*G. raimondii* and *G. arboreum*). In *G. hirsutum*, 93 STARD genes exhibited balanced subgenomic distribution: 48 localized to the Gh-A subgenome (12 chromosomes) and 45 to Gh-D (12 chromosomes), maintaining a near 1:1 allocation ratio, with no distribution observed on chromosome 4 (Figure 2). Chromosomal occupancy varied significantly, with maximum density on Gh-A05 (9 genes) and Gh-D05 (9 genes), contrasting with minimal representation on Gh-A02,09,11 and 13 (2) and Gh-D01,02,11 and 13 (2). Notably, all homologous chromosome pairs exhibited equivalent gene counts between subgenomes (Figure 2).

A parallel analysis of *G. barbadense* revealed 93 *STARD* genes positioned across 25 chromosomes, with subgenomic partitioning of 48 in Gb-A and 45 in Gb-D. Chromosomal distribution patterns mirrored those of *G. hirsutum*, with Gb-A05 harboring peak density (nine genes) versus minimal occupancy (one gene) on Gb-A02. A diploid analysis showed *G. arboreum* (48 STARDs) distributing across 11 chromosomes, while *G. raimondii* (46 STARDs) demonstrated skewed chromosomal allocation, with chromosome 9 containing 10 genes versus chromosome 13 holding only 2. A comparative subgenomic alignment between tetraploid cotton lineages and their diploid progenitors confirmed conserved chromosomal distribution patterns across evolutionary strata.

### 3.4. Collinearity Analysis and Selective Pressure Analysis

The evolution of gene families has primarily undergone three processes: whole-genome duplication or polyploidization, segmental duplication, and transposed duplication. These processes serve as the major driving forces behind the formation and evolution of gene families. Based on the open reading frame (ORF) sequences of all genes in each species, an analysis was conducted on the *STARD* family genes of four types of cotton to determine gene duplication and collinearity relationships.

**Figure 3:**
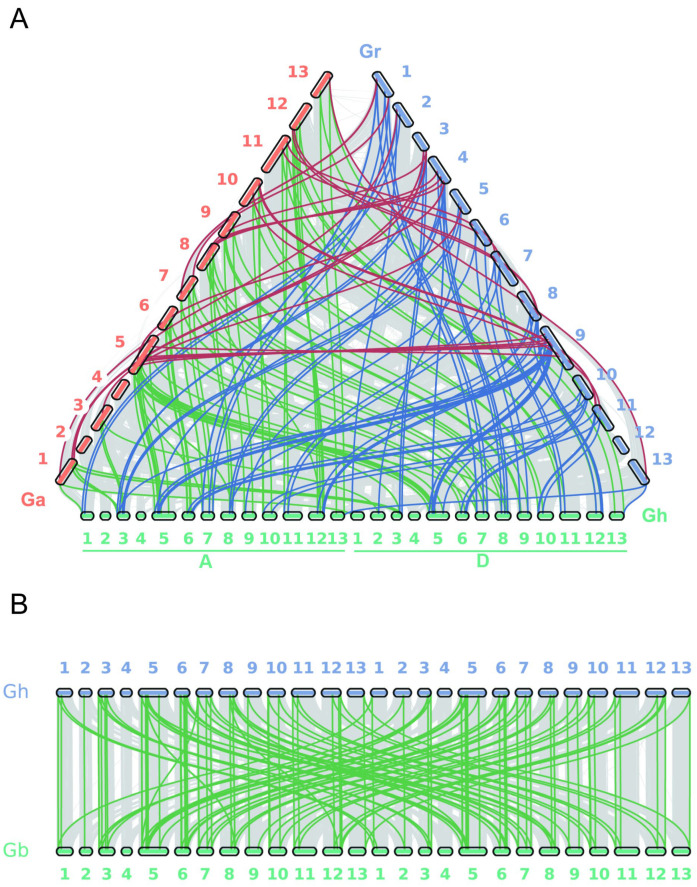
Synteny analysis of *G. hirsutum L.* with other cotton species: (**A**) synteny analysis between *G. hirsutum* and diploid cotton species *G. raimondii*/*G. arboreum*; (**B**) synteny analysis between *G. hirsutum* and allotetraploid cotton *G. barbadense*. Colored lines highlight syntenic blocks of the *STARD* gene family, while gray lines indicate syntenic regions from other genomic contexts.

In *G. hirsutum*, *G. barbadense*, *G. arboreum*, and *G. raimondii*, 129, 126, 34, and 32 pairs of genes with segmental duplication were identified, respectively. Additionally, three, two, four, and one pair of transposed duplicated genes was found in each of these four cotton species. Furthermore, one pair of tandemly duplicated genes was identified in both *G. arboreum* and *G. raimondii*. All these findings indicate that both segmental duplication and transposed duplication have played crucial roles in the expansion of STARD family proteins in *G. hirsutum*, *G. barbadense*, *G. arboreum*, and *G. raimondii*, with segmental duplication being predominantly present across these four cotton species. Moreover, the distribution of whole-genome duplicated gene pairs among these four cotton species is illustrated in Figure 3 and Table S2.

**Figure 4:**
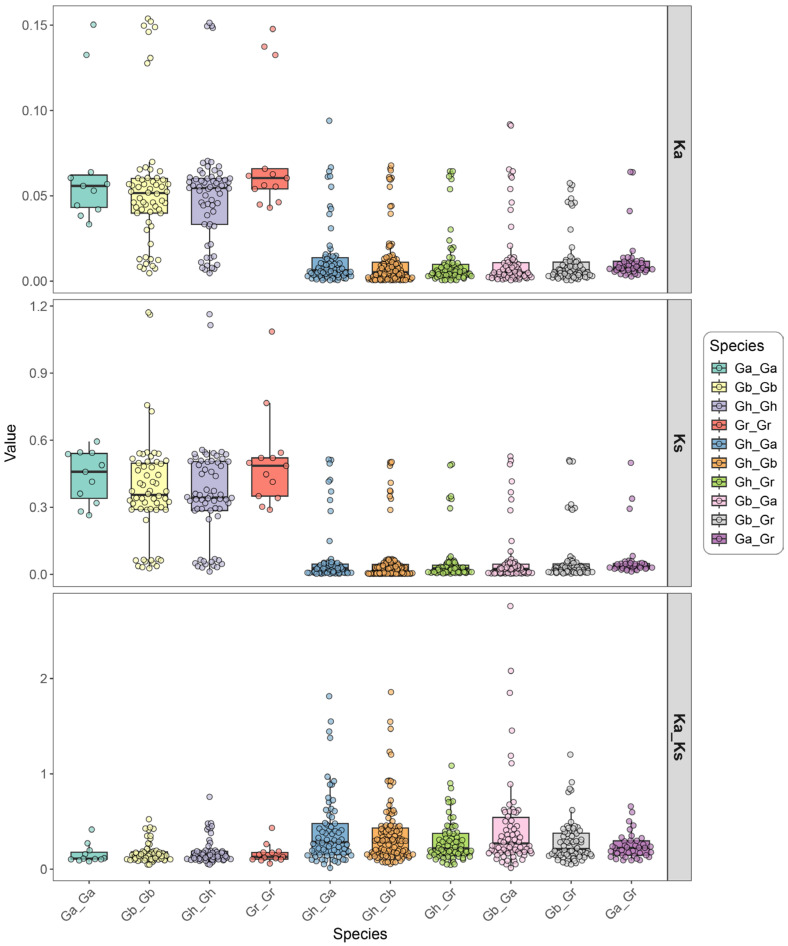
Ka and Ks analysis of *STARD* genes across cotton species. Box plots display the distribution of non-synonymous substitution rates (Ka), synonymous substitution rates (Ks), and their ratios (Ka/Ks) for different comparison groups, arranged vertically from top to bottom.

The ratio of non-synonymous (Ka) to synonymous (Ks) substitution rates (Ka/Ks) serves as a valuable metric for evaluating sequence divergence in protein-coding genes across species or taxa with uncertain phylogenetic relationships. This ratio reflects the selective pressures acting on homologous genes: Ka/Ks > 1 indicates positive selection, Ka/Ks = 1 suggests neutral evolution, and Ka/Ks < 1 implies purifying selection (Figure 4).

Within allotetraploid *Gh*, Ka/Ks values for *STARD* paralogous gene pairs ranged from 0.05–0.76, with no pairs exceeding 1. In interspecific comparisons among allotetraploid cotton species, 5/141 (3.55%) *STARD* orthologous gene pairs between *G. hirsutum* and *G. barbadense* exhibited Ka/Ks > 1, including one pair with Ka/Ks > 1.8, while the remaining values ranged from 0.01–0.99. For *G. hirsutum* vs. diploid *G. arboreum*, 5/71 (5.63%) STARD orthologs showed Ka/Ks > 1 (0–0.93 range for others), and 1/73 (1.37%) *G. hirsutum* vs. *G. raimondii* STARD orthologs exceeded Ka/Ks = 1 (0–0.90 range otherwise) (Figure 4 and Table S3).

Comparisons between allotetraploid and diploid species revealed 8/153 (5.23%) *G. barbadense* vs. *G. arboreum* STARD orthologs with Ka/Ks > 1 (0–0.97 otherwise) and 1/73 (1.37%) *G. barbadense* vs. *G. raimondii* STARD orthologs exceeding Ka/Ks = 1 (0–0.91 range). Within allotetraploid *G. barbadense*, STARD paralogs displayed Ka/Ks values between 0.05–0.52. Among diploid comparisons, *G. arboreum* vs. *G. raimondii* STARD orthologs showed Ka/Ks values from 0–0.66, while *G. arboreum* STARD paralogs ranged 0.08–0.42 and *G. raimondii* STARD paralogs ranged 0.06–0.43 (Table S3).

### 3.5. Structure Analysis of Cotton STARD Genes

**Figure 5:**
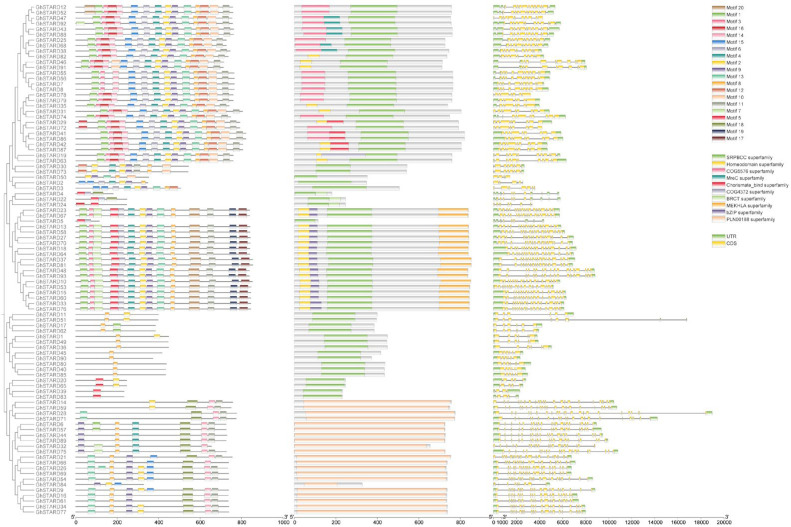
Analysis of phylogenetic relationships, protein domains, and gene structures of *GhSTARD*s. (**A**) Phylogenetic analysis of GhSTARDs proteins. Conserved protein motifs in the amino acid sequences of *GhSTARD*s were analyzed using the MEME suite. (**B**) Visualization of gene structures for *GhSTARD*s, including exon–intron organization and untranslated regions (UTRs). (C) Identification and characterization of functional domains within GhSTARD proteins.

During the evolution of gene families, the diversification of protein domains serves as the foundation for the emergence of novel protein functions, enabling organisms to adapt to environmental changes. To investigate the evolutionary dynamics of the *GhSTARD* gene family, we conducted a comprehensive analysis of protein domain architectures and conserved motifs. Using the MEME suite(Version 5.5.8 https://meme-suite.org/meme/ accessed on 10 March 2025)., we identified 20 potential motifs within *GhSTARD* members. These motifs exhibited a non-random distribution across the 93 *GhSTARD* sequences, showing variability between evolutionary clades. Clade1 predominantly contains motif1, motif3, and motif15, with most members harboring the COG5576 superfamily domain, while some additionally possess MreC superfamily or Chorismate_bind superfamily domains. Clade2 is characterized by unique motifs 17, 19, and 20, which likely underpin their distinct functional characteristics, and all members of this clade contain the MEKHLA superfamily domain. Clade3 represents the most motif-depleted group, containing only motifs 2, 5, 7, and 8. Clade4 exhibits a unique motif 18 signature and is associated with the PLN00188 superfamily (Figure 5).

Furthermore, we explored the structural diversity of *GhSTARD* genes. The exon–intron distribution pattern correlates with gene phylogeny, and our analysis revealed that *GhSTARD* exons range in length from 5 to 766 bp. While exon counts vary across clades, most *GhSTARD* members within the same clade exhibit conserved exon–intron architectures, reflecting their evolutionary relationships.

### 3.6. Cis-Element Analysis of GhSTARD Gene Promotors

**Figure 6:**
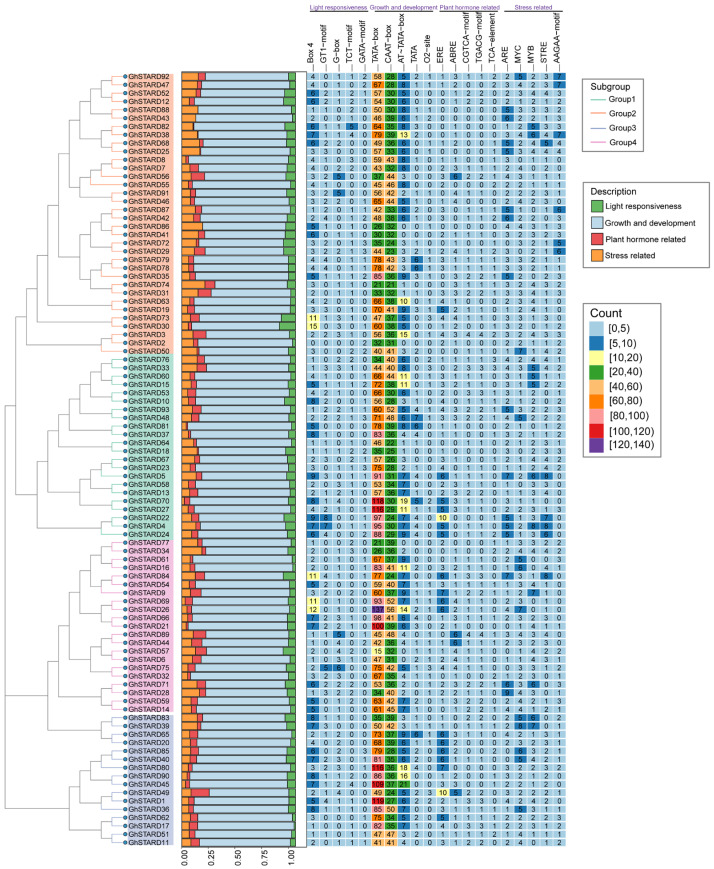
Analysis of *GhSTARD* gene promoters. From left to right: (**A**) phylogenetic analysis of GhSTARD proteins, (**B**) categorical quantification of promoter motifs, and (**C**) detailed distribution of specific promoter elements across genomic regions.

*Cis*-acting elements directly regulate the functional expression of downstream genes. In this study, the 2000 bp upstream sequences of *GhSTARD* genes were analyzed to identify *cis*-acting elements. The promoter regions contained 20 predominant types of *cis*-acting elements, which were categorized into four major classes: light responsiveness, plant growth and development, plant-hormone-related, and stress-related elements (Figure 6). Among these, TATA-box and CAAT-box (associated with plant growth and development) were the most abundant, followed by Box4 (light responsiveness) and low-temperature responsive elements. Additionally, five stress-related elements—ARE, MYC, MYB, STRE, and AAGAA-motif—were identified in the promoter regions of the GhSTARD gene family. Notably, genes in Group 4 harbored the highest number of *cis*-acting elements, whereas Group 2 exhibited the lowest average count. These findings suggest that *GhSTARD* genes may play critical roles in regulating plant growth and development, as well as in mediating abiotic stress responses.

### 3.7. The Induced Expression of Cotton STARD Genes Under Salt Stress

**Figure 7:**
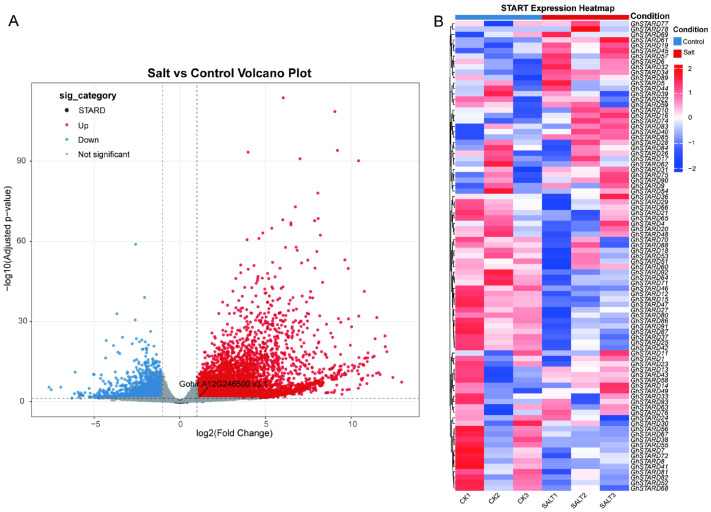
RNA-seq analysis results. (**A**) Volcano plot depicting differentially expressed genes (DEGs), with statistical significance (adjusted *p*-value) plotted against log2 fold change. (**B**) Heatmap illustrating the expression profiles of *STARD* genes across experimental conditions, with color gradients representing relative expression levels.

To elucidate the role of *STARD* genes in salt stress resistance, we conducted a differential expression analysis using published RNA-seq datasets from cotton (*Gh*) under salt stress [36]. Among the identified differentially expressed genes (DEGs), only 11 *GhSTARD* genes exhibited significant expression changes. Notably, four genes (*GhSTARD61*, *GhSTARD90*, *GhSTARD45*, and *GhSTARD85*) were upregulated in response to salt stress, while the remaining seven showed suppressed transcription levels. These findings suggest that a subset of *GhSTARD* genes specifically responds to salt stress and contributes to stress resistance. To investigate the scarcity of differentially expressed *GhSTARD* genes, we further analyzed their expression patterns using heatmap visualization. The results revealed that most *GhSTARD* genes displayed higher basal expression levels in control conditions (CK). However, limited reproducibility was observed for certain genes across biological replicates, which may partially explain the relatively small cohort of salt-responsive *GhSTARD* genes identified in this study (Figure 7).

### 3.8. Tissue Expression Patterns of GhSTARD45

**Figure 8:**
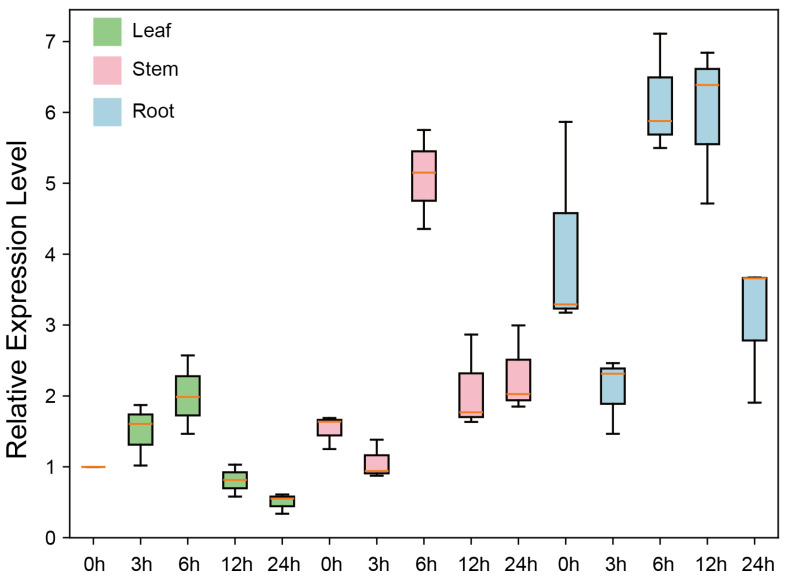
The qPCR analysis of *GhSTARD45* expression across multiple tissues under salt stress at various time points.

Among these four genes, *GhSTARD45* exhibited the highest fold change and absolute expression levels, prompting further downstream analysis. To investigate the tissue-specific expression dynamics of *GhSTARD45* under salt stress, we performed quantitative real-time PCR (qRT-PCR) on leaf, stem, and root tissues collected at 0, 3, 6, 12, and 24 h post-salt treatment (Table S4). The results revealed distinct expression profiles: leaf tissues showed consistently low *GhSTARD45* transcription, reaching minimal levels at 24 h and stem tissues exhibited moderate expression with a peak at 6 h, whereas root tissues displayed the highest transcriptional activity, with significant induction at both 6 h and 12 h timepoints. This tissue-specific induction pattern, particularly the robust upregulation in roots under salt stress, suggests that *GhSTARD45* may play a critical role in root-mediated salt tolerance mechanisms (Figure 8).

### 3.9. Gene Interaction Network

**Figure 9:**
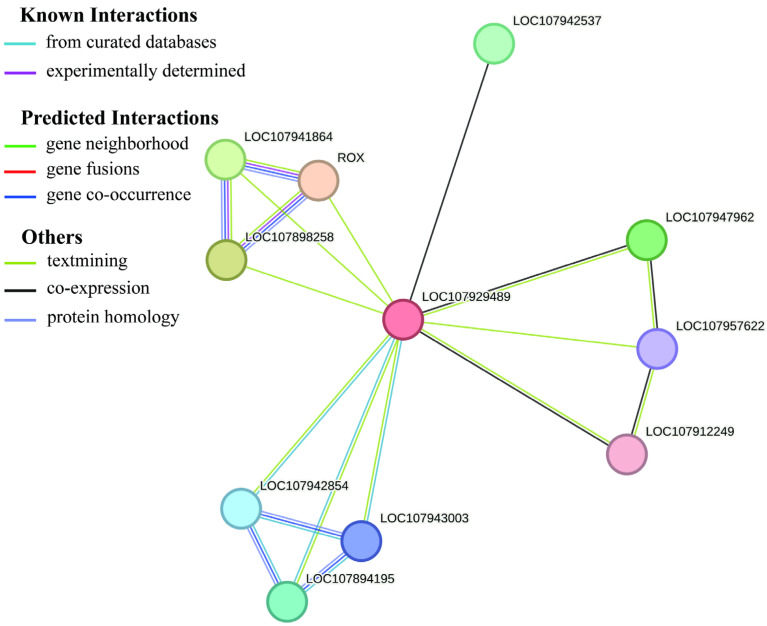
In silico prediction of protein–protein interactions (PPIs) for GhSTARD45.

This study predicted the potential protein–protein interaction (PPI) network of the cotton target gene *GhSTARD45* using multi-source bioinformatics approaches. Known interactions were derived from curated databases and experimentally validated data, while predicted interactions were generated through algorithms such as gene neighborhood, gene fusion, and gene co-occurrence. The prediction results revealed that *GhSTARD45* potentially interacts with genes including LOC107941864, LOC107898258, LOC107957622, LOC107912249, LOC107943003, and LOC107894195. Additional candidate genes, such as LOC107942537 and LOC107947962, were also incorporated into the interaction network. Functional annotation indicated that these genes encode bHLH140 and probable 1-acyl-sn-glycerol-3-phosphate acyltransferase, respectively, suggesting that the target gene may participate in complex regulatory pathways. These findings provide critical insights for future functional validation and molecular mechanism studies in cotton (Figure 9).

## 4. Discussion

STARD genes have been categorized and described across various plant species [37]. Previous studies have identified 35 and 25 STARD gene members in Arabidopsis thaliana and rice, respectively, while only 23 STARD genes were identified in grapevine [19]. This study identified a significantly expanded STARD gene family (46–93 members) in tetraploid cottons, likely resulting from ancient and recent whole-genome duplications. We propose asymmetric subgenome-specific retention as a potential mechanism underlying this expansion. Notably, differential STARD gene retention between A/D subgenomes in *G. hirsutum* and *G. barbadense* suggests divergent adaptive contributions from ancestral genomes post-allopolyploidization. We speculate that this might be attributed to the presence of a START domain in the proteins of the HD-Zip III and HD-Zip IV subgroups, which is involved in processes such as cell differentiation, apical meristem differentiation, embryonic development, microtubule formation, morphogenesis, auxin polarity transport, and responses to biotic and abiotic stresses [7,9,38,39,40]. These processes are closely related to the growth characteristics of cotton.

Current research indicates that it is crucial to note that cultivated allotetraploid cottons, *Gh* and *Gb*, generally exhibit superior tolerance to salt, drought, and oxidative stress compared to their diploid relatives (*G. arboreum* and *G. raimondii*). This key phenomenon warrants in-depth exploration of its underlying molecular mechanisms in the discussion. Under salt stress, the *G. barbadense* cultivar Hai7124 outperformed the *G. hirsutum* accession TM-1, activating stronger defense-related responses (e.g., antioxidant metabolism and ABA/JA signaling pathways), while TM-1 prioritized pathways associated with ion homeostasis and growth. Furthermore, although STARD genes are widely present, their specific role in the salt stress response remains largely uncharacterized [41,42,43,]. A previous study in grapevine identified 23 *STARD* genes, with the majority exhibiting significant upregulation under NaCl treatment, while the remaining genes showed induced expression following methyl jasmonate (MeJA) treatment [19]. In contrast, our findings in cotton (*Gh*) reveal a distinct regulatory pattern: the majority of *GhSTARD* genes exhibited non-significant differential expression under salt stress, and differentially expressed members were predominantly downregulated. These observations suggest that while *STARD* genes may contribute to salt tolerance in cotton, they likely represent secondary regulatory components rather than primary mediators. Furthermore, although this study systematically characterized four candidate *GhSTARD* genes through bioinformatics analyses and identified salt-responsive candidates from RNA-seq datasets, functional validation via molecular experiments remains essential.

The protein–protein interaction (PPI) analysis predicted that GhSTARD45 interacts with a basic helix–loop–helix (bHLH) transcription factor. Notably, the promoter analysis revealed the significant enrichment of *cis*-elements associated with MYB transcription factors [44,45,46]. Given that MYB, bHLH, and WD40 proteins form a ternary complex (MYB-bHLH-WD40 and MBW complex) to co-regulate target gene expression [45], we hypothesize that this identified bHLH gene may function as a critical regulatory component upstream of GhSTARD45. Furthermore, potential candidate MYB transcription factors could be prioritized through integrated approaches such as co-expression network analysis, leveraging the known functional synergy between these transcription factor families in biological regulatory networks. Collectively, these results establish a foundation for investigating salt tolerance mechanisms in cotton and provide a theoretical basis for prioritizing gene candidates in future research.

While the identification of *GhSTARD45* as a salt-responsive gene is promising, we acknowledge that the current study lacks deeper functional validation, such as transgenic overexpression, CRISPR knockouts, or phenotypic assays. This limitation restricts the strength of our functional claims. Future research should focus on these areas to confirm the role of *GhSTARD45* in salt tolerance. For instance, transgenic cotton lines overexpressing *GhSTARD45* could be generated and assessed for salt tolerance phenotypes. Similarly, CRISPR/Cas9 technology could be employed to create knockout mutants, and the resulting plants could be analyzed for alterations in salt stress responses. These experiments would provide more direct evidence of *GhSTARD45*′s function and its potential application in improving cotton salt tolerance.

## 5. Conclusions

This study provides novel insights into the role of the cotton *STARD* gene family in salt tolerance. Our findings reveal that the expansion of the *STARD* gene family in tetraploid cottons is primarily driven by whole-genome duplications, with asymmetric retention between A/D subgenomes. While most *GhSTARD* genes show no significant differential expression under salt stress, specific genes like *GhSTARD45* are significantly upregulated in root tissues, suggesting their potential role in salt tolerance. Additionally, the protein–protein interaction network analysis of *GhSTARD45* indicates its involvement in complex regulatory pathways. These results offer new perspectives for enhancing cotton salt tolerance through genetic engineering and provide valuable genetic resources for future research.

## Data Availability

The original contributions presented in the study are included in the article, and further inquiries can be directed to the corresponding author.

## Supplementary Materials

The following supporting information can be downloaded at https://www.mdpi.com/article/10.3390/genes16070813/s1 Table S1: Characteristic information of the *STARD* gene; Table S2: Gene duplication information of cotton STARD; Table S3: Analysis results of *STARD* gene Ka and Ks.
